# The effect of lactulose supplementation on fecal microflora of patients with chronic kidney disease; a randomized clinical trial

**DOI:** 10.15171/jrip.2016.34

**Published:** 2016-07-29

**Authors:** Hamid Tayebi-Khosroshahi, Afshin Habibzadeh, Bahram Niknafs, Reza Ghotaslou, Fatemeh Yeganeh Sefidan, Morteza Ghojazadeh, Majid Moghaddaszadeh, Sahar Parkhide

**Affiliations:** ^1^Kidney Research Center, Tabriz University of Medical Sciences, Tabriz, Iran; ^2^Medical Education Research Center, Tabriz University of Medical Sciences, Tabriz, Iran

**Keywords:** Chronic kidney disease, Lactulose, Fecal microflora, Bifidobacteria, Lactobacilli

## Abstract

**Introduction:** Lactulose is a prebiotic with bifidogenic and urea reduction effects. It can improve Bifidobacteria and Lactobacilli counts in healthy humans and it may possibly have similar effects in chronic kidney disease (CKD) patients.

**Objectives:** To investigate the effect of lactulose on fecal microflora of patients with CKD.

**Patients and Methods:** Thirty-two patients with stages 3 and 4 of CKD (43.8% male with mean age of 58.09±12.75 years) were randomly assigned to intervention (n=16) and control (n=16) groups. Patients in intervention group received 30 mm lactulose syrup three times a day for an 8-week period. Control group received placebo 30 mm three times a day. A fecal sample was obtained from all patients at the beginning and at the end of the study and Bifidobacteria and Lactobacilli was counted.

**Results:** Creatinine (Cr) significantly decreased in intervention group (3.90±1.43 to 3.60±1.44, *P*=0.003) and increased in control group (3.87±2.08 to 4.11±1.99, *P*=0.03). Although Bifidobacterial and Lactobacilli counts were similar before intervention, they were significantly higher at the end of the study in lactulose group (*P*=0.01 and *P*=0.04, respectively). Lactulose led to significant increase in fecal Bifidobacterial counts (3.61±0.54 to 4.90±0.96, *P*<0.001) and Lactobacilli counts (2.79±1.00 to 3.87±1.13, *P*<0.001), while the change in placebo group was not significant.

**Conclusion:** Lactulose administration will increase Bifidobacteria and Lactobacillus counts in patients with CKD.

**Implication for health policy/practice/research/medical education:**

Lactulose as a prebiotic can improve Bifidobacteria and Lactobacilli counts. The aim is the evaluation of lactulose on intestinal flora of chronic kidney disease patients. We used lactulose and compared the bifidobacteria and lactobacillus colony counts and also nitrogenous waste products with control patients. There was significant reduction of urea, creatinine in improvement of Bifidobacteria and Lactobacillus counts.

## Introduction

In the last decades, considerable efforts have been directed at improving human (as well as animal) health or preventing disease by the use of functional foods to which prebiotics and probiotics belong (1,2). Prebiotics are non-digestible and selectively fermented food ingredients that modulates beneficial gastrointestinal microflora, both in the composition and/or their activities, thus generating benefits to human health. Lactobacilli and Bifidobacteria are the usual target genera for prebiotics (3,4). Numerous scientists investigated the health-promoting effect of prebiotics like indigestible sugars, e.g. fructooligosaccharides, inulin and lactulose (5,6).

The positive effects of lactulose on colonic metabolism in human is well known (7). Lactulose is a commercially available disaccharide that is used as a drug in the treatment of hepatic encephalopathy and chronic constipation (8,9), which has been shown to stimulate the growth of Bifidobacteria (10).

Uremia is an illness that accompanies renal failure and chronic kidney disease (CKD). Uremic illness is considered to be due largely to the accumulation of organic waste products that are normally cleared by the renal. However, uremic products are also generated in the gastrointestinal tract (GIT). Toxins generated in, or introduced into the body via the intestine, like advanced glycation end products, phenols, and indoles, all may contribute to the pathogenesis of CKD. Another problem in CKD patients is the prolonged colonic transit time that promotes uremia retention and absorption. It is supposed that prebiotics like lactulose have a therapeutic role in maintaining a metabolically balanced GIT with increased beneficial GIT microflora, and would reduce progression of CKD and associated uremia (11-13). In CKD, lactulose could promote fecal excretion of water, sodium, potassium, ammonium, urea, creatinine (Cr) and protons (14). In our previous study, we showed that lactulose administration in CKD patients could decrease levels of various deleterious elements, especially nitrogen products (15).

Due to the role of lactulose as a prebiotics in GIT and its possible positive effects on CKD patients, we aim to evaluate the effects of lactulose supplementation on fecal microflora in these patients.

## Patients and Methods

### Study population

**Figure 1:**
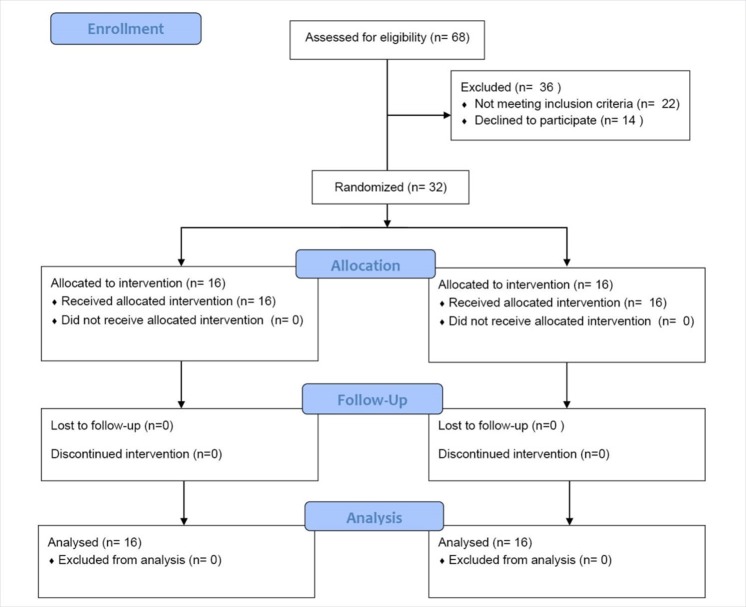
Patients who allocated to receive lactulose and control group.

In this randomized controlled clinical trial, 32 patients with CKD in the stages 3 and 4 were recruited and randomly assigned to intervention (n=16) and control (n=16) groups. During the study no patients were excluded or withdrawn in any groups (Figure 1). Patients over 18 years old with an estimated glomerular filtration rate (eGFR) ≤60 ml/min/1.73 m^2^ and stages 3 and 4 of CKD, with no history of gastrointestinal or metabolic disease or previous surgery (apart from appendectomy), and without antibiotic treatment or any other medical treatment influencing intestinal microbiota especially probiotics, prebiotics and symbiotics during the 3 months before the start of the study were included. Subjects were advised to maintain their usual diet during the study period and to avoid the intake of fermented milk products and food components containing high quantities of fermentable carbohydrates. The treatment would be stopped if any major complications would have happened and if any patient had diarrhea due to lactulose use, the treatment would be stopped and after a period start with lower dosage. Those could not keep up with the study protocol or with drug intolerance were excluded. The subjects did not have to restrict their everyday diet, medication or daily activities. Those drugs which induce constipation such as AST-120, polystyrene sulfonate and potassium binders were not administered.

### Study protocol

The study was conducted over an 8-week period. Patients in case group received 30 mm lactulose syrup (Zahravi Co, Tabriz, Iran) three times a day. The doses administered were chosen based on therapeutic recommendations for CKD patients in a way that subjects did not suffer from negative effects or discomfort. Control group received placebo 30 mm three times a day. All patients were visited weekly in order to evaluate the possible side effects and assessing their adherence to medication. The same company prepared a placebo solution with the same color and taste.

### Fecal analysis

A fecal sample was obtained from all patients at the beginning and the end of the study. They were collected in plastic containers under anaerobic conditions, immediately stored at 4°C and analyzed within 3 hours. Stool samples were homogenized with a high-speed blender and serially diluted 10-fold in solution for anaerobes (saline, glucose and cysteine). A volume of 100 mL of each dilution was inoculated in appropriate agars. Bifidobacteria and Lactobacilli were cultured for respectively 5 and 3 days at 37°C in anaerobic conditions (Gas Pak A system, BBL, Cockeysville, MD, USA) with Anaerocult A. Bifidobacteria were counted on the Beerens medium (Beerens, 1991); Lactobacilli were counted on the Rogosa medium.

### Ethical issues

The research followed the tenets of the Declaration of Helsinki. Informed consents were obtained from parents and the research was approved by the Ethics Committee of Tabriz University of Medical Sciences. This clinical trial had a registration ID: IRCT201105302858N2 in Iran (http://www.irct.ir/).

### Statistical analysis

Statistical analyses were performed using the SPSS version 16.0 (SPSS Inc., Chicago, Illinois). Faecal concentrations of bacteria were expressed as log colony forming unit (CFU)/g wet weight. Continuous values were expressed as mean ± stan­dard deviation (SD) and categorical variables were expressed as percentages. The categorical parameters were compared by χ^2^ tests or Fisher’s exact test. The continuous variables before and after intervention between groups was compared with independent *t* test and within each group was compared with paired samples *t* test. A *P* value less than 0.05 was considered significant.

## Results

Thirty-two CKD patients (18 female with mean age of 58.09 ± 12.75 years) were divided into intervention (n=16) and control (n=16) groups. Patients’ baseline findings are demonstrated in Table 1. There was no difference in baseline findings between groups.

**Table 1:** Patients’ baseline findings

	**Intervention**	**Control**	***P*** ** value**
Age (y)	59.31 ± 12.97	56.88 ± 12.84	NS
Gender, male, n (%)	9 (56.3%)	5 (31.3%)	NS
Etiology of CKD			
Diabetes mellitus	4	8	
Hypertension	6	4	
Glomerulonephritis	1	2	
Polycystic kidney disease	2	0	
Unknown	3	2	

Abbreviations: CKD, Chronic kidney disease; NS: not significant

Table 2 demonstrates the laboratory findings before and after intervention in both groups. There were no significant differences between groups in hemoglobin (Hb), Cr and blood urea nitrogen (BUN) before and at the end of the study. Comparing the results before and at the end of the study within each group, we observed that Cr significantly decreased in intervention group (from 3.90±1.43 to 3.60±1.44, *P*=0.003). In control group receiving placebo, Hb was significantly decreased (from 11.080.82 to 10.93±0.92, *P*=0.02) and Cr was significantly increased (from 3.87±2.08 to 4.11±1.99, *P*=0.03). The percentage of change in Cr level during the trial was calculated and showed that in intervention group Cr was decreased while it was increased in control group and the difference was significant (-8.01±2.40 versus 10.20±4.94; *P*=0.002).

**Table 2:** Laboratory findings before and after intervention in both groups

	**Intervention**	**Control**	***P*** ** value**
Hb (g/dL)			
Before	11.00 ± 1.36	11.08 ± 0.82	NS
At the end	11.16 ± 1.19	10.93 ± 0.92	NS
Cr (mg/dL)			
Before	3.90 ± 1.43	3.87 ± 2.08	NS
At the end	3.60 ± 1.44	4.11 ± 1.99	NS
BUN (mg/dL)			
Before	55.31 ± 14.54	53.88 ± 14.68	NS
At the end	48.38 ± 15.54	55.38 ± 11.98	NS

Table 3 demonstrates the effect of 8-week ingestion of lactulose or placebo (30 mL/d) on the fecal bacterial counts in CKD patients. Before intervention, there were no significant differences between groups in fecal Bifidobacterial counts and Lactobacilli counts. At the end of the study, fecal Bifidobacterial counts and Lactobacilli counts were significantly higher after lactulose consumption compared to placebo ingestion. Lactulose led to significant increase in fecal Bifidobacterial counts (*P*<0.001) and Lactobacilli counts (*P*<0.001). Placebo consumption in control group did not lead to any significant fecal Bifidobacterial (*P*=0.13) or Lactobacilli (*P*=0.15) count change.

**Table 3:** Effect of 8-week ingestion of lactulose or placebo (30 mL, three times a day) on the fecal bacterial counts (mean ± SD; log CFU/g) in CKD patients

	**Intervention**	**Control**	***P*** ** value**
Bifidobacterial counts (log CFU/g)			
Before	3.61 ± 0.54	3.81 ± 0.70	0.37
At the end	4.90 ± 0.96	4.03 ± 0.90	0.01^a^
Lactobacilli counts (log CFU/g)			
Before	2.79 ± 1.00	3.14 ± 1.28	0.39
At the end	3.87 ± 1.13	3.04 ± 1.14	0.04^a^

^a^ *P* is two sided significant.

There were not any considerable side effects regarding lactulose use.

## Discussion

In this randomized clinical trial we evaluated the effects of lactulose on fecal microflora in CKD patients and observed significant increase in fecal bifidobacterial and lactobacillus count in patients receiving lactulose, which showed no difference in placebo group.

There are no studies evaluating lactulose effects on GIT microflora in CKD patients. Previous studies on healthy humans have shown significant increase in fecal bifidobacteria counts in healthy and even in cirrhotic patients and those with idiopathic constipation (10,16,17). However, unlike our findings, none of these studies could show any significant changes in lactobacillus counts. It is shown in other prebiotics except lactulose that Lactobacilli count significantly increases after prebiotic administration (18,19). In contrast to aforementioned results, Bouhnik et al (20) observed that lactulose was not bifidogenic and did not cause significant increase in the bifidobacteria count; however the study was performed on healthy subjects and in duration of 8 days which would affect the results.

Accumulation of urea in the body fluids in humans and animals with renal failure leads to its heavy influx into the GIT (21), which is compounded by microbial colonization of the upper intestinal tract and dramatic change in the composition of the gut microbiome (22).This microbial colonization in intestine in CKD patients are mostly due to inefficient protein assimilation in the small intestine resulting in more protein entering the large intestine, prolonged colonic transit time, and increased luminal pH secondary to increased colonic urea diffusion (11). As well it is demonstrated that colonic microbial activity may contribute to uremic solute production (23). New studies are aim to find a way to reduce unwanted bacterial colonization by increasing beneficiary microflora including bifidobacteria and lactobacillus. This improvement could produce compounds to inhibit potential pathogens, produce digestive enzymes and reduce blood ammonia levels and constipation (24).

The prebiotic lactulose has the potential to alter fecal flora (25). It is neither absorbed nor metabolized in the upper GIT but is degraded to organic acids by bacteria of the proximal colon (26). Lactulose mostly can act as a substrate for many lactic acid bacteria and increase their amount mostly Bifidobacteria and Lactobacilli. It also decreases significantly the number of (lecithinase-positive) clostridia and Bacteroidaceae decreased (27). However, we only evaluated the changes in the amount of Bifidobacteria and Lactobacilli.

The low concentrations of Lactobacilli and Bifidobacteria have been shown previously in renal failure patients (28,29). It is suggested that prebiotic intake may be particularly effective for subjects exhibiting low intrinsic numbers of Bifidobacterial (30,31). It is possible that prebiotics like lactulose be also effective in subjects with lower Lactobacilli count. CKD patients in our study had very lower Bifidobacteria and Lactobacilli counts than healthy subjects reported in the literature which was significantly increased after lactulose administration.

Elevated levels of p-cresol have recently been demonstrated to be correlated to a higher mortality in uremic syndrome (32). The exact pathogenic mechanisms occurring in case of kidney disease are not completely understood today. Therefore, strategies that counteract the accumulation of p-cresol and other protein fermentation metabolites might constitute a significant improvement in the management of those patients. De Preter and colleagues observed a significant reduction of the urinary 15N and p-cresol (as a part of uremic toxins) excretion after the intake of lactulose, which was accompanied by a significant increase in the fecal 15N output (33,34). These studies indicate that subjects with higher baseline levels of uremic toxins would show a higher response to prebiotic dosing.

The mechanism of increasing bifidobacteria and lactobacillus after lactulose administration is ambiguous. There are two considerable processes. One possibility is lactulose increased directly bifidobacteria and lactobacillus, consequently it declined serum Cr and BUN level. Another is lactulose reduced uremic toxins, so it declined serum Cr and BUN level, consequently it increased bifidobacteria and lactobacillus.

To consider the second possibility, we need to measure uremic toxins. We only evaluated blood levels of BUN and Cr and observed a significant reduction in Cr levels after lactulose administration, which had a significant increase in placebo group. Although not measuring the uremic toxins in our study could be a great limitation to our findings; it could be concluded that lactulose could reduce uremic toxins; however, the exact mechanism and effect should be evaluated using p-cresol and other uremic toxins.

As mentioned, we only observed significant decrease in Cr levels in the intervention group and Cr increase in control group with no significant difference in BUN levels. However, in our previous study, we observed significant decrease in urea and Cr after treatment with lactulose (15). In another study, Miranda Alatriste and colleagues (35) only observed 11% reduction in BUN levels of CKD patients on probiotic supplementation. They concluded that lower dosage of supplementation, amount of protein consumption and small sample size can affect the result. It is possible that similar factors have role in the low non-significant decrease of BUN in our study.

We also observed that Hb was maintained in the intervention group while had significantly decreased in the control group. Cetin and colleagues (36) observed significant increase in RBC counts, Hct and Hb values in the group supplemented with probiotic compared with control. Such increase has also been reported in animal studies (37). However, in our previous study we observed no significant difference between Hb values before and after treatment (15).Considering these findings, it is possible that dietary probiotic and prebiotic supplementation such as lactulose may improve Hb levels in normal subjects and even in CKD patients.

This study as the first study evaluating lactulose effects in CKD patients has some strengths and limitations; this is a randomized clinical trial with control group that allowed a better comparison of the results between groups and better evaluation of the lactulose efficacy. As a limitation we did not measure uremic toxins and could not evaluate the exact effects of lactulose on reducing these toxins by influencing the GIT. However, the reduction in the Cr levels and not increase in its values could be the effect of this prebiotic in this regard.

## Conclusion

The results of current study showed that lactulose administration will increase bifidobacteria and lactobacillus counts in patients with CKD. Considering our previous findings regarding the role of lactulose in reducing the uremic toxins, it is possible that increase bifidobacteria and lactobacillus counts can cause reduction of uremic toxins and also the BUN and Cr which would improve the function of the kidney in CKD patients.

## Limitations of the study

The limitation of this study was small proportion of patients. Thus we suggest more investigations on this aspect of kidney diseases patients.

## Acknowledgments

The authors wish to thank the hemodialysis staffs of Shahid Madani and Imam-Reza hospitals for their continuing efforts.

## Authors’ contribution

HTK and BN conceived the study and contributed reagents and tools. RG and MM performed the study. AH, MG and SP analyzed the data and drafted the final manuscript. All authors read, revised and approved the final manuscript.

## Conflicts of interest

The authors report no conflicts of interest. The authors alone are responsible for the content and writing of the article.

## Ethical considerations

Ethical issues (including plagiarism, data fabrication, double publication) have been completely observed by authors.

## Funding/Support

This study was extracted from residential thesis of Bahram Niknasfs and financially supported by Tabriz University of Medical Sciences (Grant# 89/3-1/1).
